# Decision curve analysis apropos of choice of preferable treatment positioning during breast irradiation

**DOI:** 10.1186/s12911-019-0927-4

**Published:** 2019-10-29

**Authors:** Ferenc Rárosi, Krisztina Boda, Zsuzsanna Kahán, Zoltán Varga

**Affiliations:** 1 Department of Medical Physics and Informatics, University of Szeged, Korányi fasor 9, Szeged, 6720 Hungary; 2 Department of Oncotherapy, University of Szeged, Korányi fasor 12, Szeged, 6720 Hungary

**Keywords:** Decision curve, Regression model, Prediction, Validation, Left-sided breast radiotherapy, LAD mean dose

## Abstract

**Background:**

Radiotherapy is a standard treatment option for breast cancer, but it may lead to significant late morbidity, including radiation heart damage. Breast irradiation performed individually in the supine or prone position may aid in minimizing the irradiation dose to the heart and LAD coronary artery. A series of CT scans and therapy plans are needed in both positions for the ‘gold standard’ decision on the preferable treatment position. This method is expensive with respect to technology and physician workload.

Our ultimate goal is to develop a predictive tool to identify the preferable treatment position using easily measurable patient characteristics. In this article, we describe the details of how model building and consequently validation of the best model are done.

**Methods:**

Different models were used: both logistic regression and multiple linear regressions were used to estimate the LAD mean dose difference (the difference between the mean dose to the LAD in the supine position versus prone position); predicted dose differences were analysed compared to the ‘gold standard’ values, and the best model was selected accordingly. The final model was checked by random cross-validation. In addition to generally used measures (ROC and Brier score), decision curves were employed to evaluate the performance of the models.

**Results:**

ROC analysis demonstrated that none of the predictors alone was satisfactory. Multiple logistic regression models and the linear regression model lead to high values of net benefit for a wide range of threshold probabilities. Multiple linear regression seemed to be the most useful model. We also present the results of the random cross-validation for this model (i.e. sensitivity of 80.7% and specificity of 87.5%).

**Conclusions:**

Decision curves proved to be useful to evaluate our models. Our results indicate that any of the models could be implemented in clinical practice, but the linear regression model is the most useful model to facilitate the radiation treatment decision. In addition, it is in use in everyday practice in the Department of Oncotherapy, University of Szeged, Hungary.

## Background

Radiotherapy is an effective treatment for breast cancer, but it can lead to significant late morbidity, particularly connected to various heart diseases [1, 2]. The goal of radiotherapy is to achieve a good therapeutic ratio, i.e. a sufficient dose in the planning target volume but as low as possible to normal tissues and especially to critical organs at risk (OAR). Great efforts are made to minimize the irradiation dose to the heart in general; nevertheless, the dose to the LAD is of the utmost clinical importance [3]. There are different approaches to protect the heart from radiation, among which breathing control, including the deep inspirational breast hold technique and IMRT or partial breast irradiation, have just recently become available in routine practice [4, 5]. Originally, prone positioning was invented for the irradiation of difficult cases with large breasts, and many groups came to favour its wide use [6–11]. Numerous studies including ours have demonstrated that the preferable position varies from patient to patient [6–8, 12–16]. Some investigations have identified patient-related predictors in favour of a particular treatment position, while others have not.

There are studies that prefer the prone position for heart protection in general; others did not find a relevant benefit of prone positioning compared to supine positioning [8, 16]. Investigations have demonstrated that the favourable treatment set-up varies from patient to patient but have not shown patient-related characteristics to predict the favourable treatment position [8, 9, 15, 16]. Others have shown an association between breast size and the benefit of prone positioning [8]. In our prospective clinical experiment, we found a strong relationship between the dose to the LAD and heart and some patient-related characteristics, such as BMI and the geography of the breast, heart and chest wall [6, 7].

Prediction models are widely used in biomedical research and other interdisciplinary fields of research. These models are mainly based on a regression method: if the dependent variable is continuous, then a multiple regression model can be used, while logistic regression is applied for categorical (often binary) dependent variables. The result of a regression model is an expected value of the dependent variable, and the result of a (binary) logistic regression is an expected probability. When the purpose is to make a decision and make predictions concerning the existence of a phenomenon, such as an illness or the necessity of an operation, the decision is based on a carefully chosen cut-point.

There are several measures to describe the performance of the prediction model, with the ROC curve being the most commonly used one [17, 18]. Unfortunately, it has the disadvantage of equally weighting false positive and false negative decisions, whereas it may be important to weight the different types of misclassifications differently.

Vickers has published a method which not only eliminates this weakness in the ROC method, but also introduces a completely different approach to derive a measure called ‘net benefit’ to evaluate the clinical utility of the method [19]. The value of the net benefit depends on the cut-point chosen. We can obtain the decision curve if we plot the net benefit in function of the cut-point (threshold probability). Decision curves can be effectively used to compare different prediction methods and to determine the range of the possible cut-point, where the use of the prediction model is beneficial. We report on how the decision curve method has been applied to evaluate different models to facilitate individualized breast irradiation.

A series of CT scans and therapy plans in both positions (supine and prone) are called for to compare the dose to the heart and LAD and to select the preferable treatment position; we call this optimization process the ‘gold standard’ decision. This method is expensive (with respect to both the technology and physician workload) and involves an extra dose of radiation to the patients. This was the motivation for creating a model to predict the preferable position and anticipate the dose difference between the two positions using the patient-related characteristics noted above. In fact, this model is already being implemented in routine radiotherapy practice at the Department of Oncotherapy, University of Szeged, Szeged, Hungary; in this article, we describe the details of how model building and consequently validation of the best model took place [6**,** 7**]**.

## Methods

### Description of data

Various patient-related features and dosimetry data extracted from radiation treatment plans generated in both prone and supine positions of 83 left-sided breast cancer cases receiving postoperative whole-breast radiotherapy were used for analyses. The details of the IRB-approved clinical study have been described elsewhere [6]. LAD and heart doses were related most strongly to body mass index (BMI) and the geography of the heart and breast. The latter could be characterized by the median distance between the LAD and the chest wall (d_median_), and the heart area included in the radiation field on a single CT scan at the middle of the heart in the supine position (A_heart_). The effect of some other measures, such as the traditionally considered breast size (corresponding to PTV), waist and hip circumferences, were less important [6]. In another 55 cases, these preliminary observations showed great consistency. Detailed evaluation was carried out on a total of 138 cases in this study. Although both the LAD and heart doses were considered in the model, since the dose to the LAD has the greatest impact on clinical decision making, this parameter is presented as the only dependent variable here.

We considered the prone position preferable if the LAD dose was smaller in that position than in the supine position, and we defined the supine position as preferable if the opposite was true. The dependent variable was the difference in the mean dose to the LAD (‘LAD mean dose difference’), meaning the mean dose to the LAD in the supine position minus the mean dose to the LAD in the prone position as derived from the radiation treatment planning system. In the event of a positive LAD mean dose difference, the prone position seemed more favourable, while the supine position was more advantageous in the event of a negative LAD mean dose difference. The LAD mean dose difference is a continuous dependent variable, but the decision is binary. The ‘gold standard decision’ on the treatment position is based on the value of the LAD mean dose difference.

### Prediction models

Our early models were logistic regression-based models using patients’ characteristics noted in the ‘Description of data’ section. The continuous dependent variable had to be dichotomized for the logistic regression-based models. We coded it 0 if the prone position was preferred and 1 if the supine position was preferred, based on the gold standard method discussed in detail above. A hierarchical cluster analysis was performed with a similarity measure of the Pearson correlation coefficient to avoid multicollinearity between independent variables. The correlations were explored between the predictors, with poorly correlated predictors chosen for model building. We were searching for a parsimonious model with relatively few and uncorrelated predictors. We applied the backward and forward likelihood ratio selection methods to select a logistic regression model.

The dependent variable was originally continuous in our dataset; the use of binary logistic regression would have led to a loss of information. Treating the dependent variable as a continuous variable, we used a multiple linear regression. Higher differences are taken into account with more weight in the regression model. Another advantage of using multiple linear regression is that the expected value of the LAD mean dose difference can also be calculated. The decision was based on the sign of the estimated dependent variable in the regression model.

It is well known that the results of the classifications are overly optimistic when the classification is made on the same dataset where the classification rule was discovered (i.e. the training set and the test set are the same). To identify the best model, we used an internal validation method of data splitting as follows: the sample was divided into two parts randomly, with 70% of the sample as the training set for the linear regression and the resultant model being tested on the remaining 30% of the data. The classification results and the misclassified dose were also noted. The process was repeated 1000 times randomly, and it was not just the proportions of misclassified patients that were taken into account, but also the distribution of the misclassified dose.

The calculations were carried out in IBM SPSS version 24 and R (version 3.3.1).

### ROC analysis

The performance of a prediction model can be evaluated by comparing the decision to the gold standard method. In most cases, we simply do not know the ‘truth’. The nearest we have to it is the gold standard, so we have to regard that as the ‘truth’. There are several measures to describe the performance of the prediction model based on the numbers in TP (true positive), FP (false positive), TN (true negative) and FN (false negative) cases. The well-known measures are sensitivity, specificity, positive predictive value, negative predictive value, accuracy (proportion of all correct diagnoses) and the Youden index (i.e. sensitivity+specificity-1). The method involving ROC curves is based on the measures noted above: we plot the sensitivity in function of 1-specificity at various threshold levels. We can also choose the optimal cut-point based on these measures and methods. One of the most frequently used methods for cut-point selection is to find the maximum value of the Youden index, with the value that maximizes the Youden index being the cut-point. Other methods for choosing a cut-point have been published [17, 18, 20–22].

### Brier score

The Brier score, originally introduced by Glenn W. Brier, is calculated as follows:

1 $$ BS=\frac{1}{N}\sum \limits_{i=1}^N{\left({p}_i-{o}_i\right)}^2 $$

and the formula for the weighted Brier score is:

2 $$ wBS=\frac{\sum \limits_{i=1}^N{w}_i{\left({p}_i-{o}_i\right)}^2}{\sum \limits_{i=1}^N{w}_i} $$

where *o*_*i*_ is the i^th^ outcome, *p*_*i*_ is the i^th^ predicted probability and *w*_*i*_ is the weight of the i^th^ item. If the outcome occurs in the i^th^ case, then *o*_*i*_ *= 1*; otherwise, *o*_*i*_ *= 0*. The Brier score measures an average (or weighted average) of squared distances between current outcomes and predicted probabilities, while lower values indicate better predictive performance [23].

The performance of the three best predictors (BMI, area and median distance) was evaluated by univariate logistic regression. For example, univariate logistic regression was employed with BMI as the independent variable (and the preferable treatment position as the dependent variable) to construct a probability prediction based on the value of BMI.

### Net benefit and decision curves

Decision curve analysis is a relatively new method to evaluate the performance of diagnostic tests and prediction models. It is based on the predicted probabilities of statistical models. The decision curve method was introduced by Vickers, based on the ‘net benefit function’ [19]. A decision curve is a plot which shows the net benefit calculated at various threshold levels. The definition of net benefit is based on the ‘utility of the prediction of the method’, originally defined by Peirce as:

3 $$ B=\frac{p\cdot TP-l\cdot FP}{TP+ FP+ FN+ TN}=\frac{p\cdot TP-l\cdot FP}{N} $$

where *p* stands for the ‘profit’ of a true positive decision, *l* refers to the ‘loss’ of a false positive decision, *TP*, *FP*, *TN* and *FN* are the number of true positive, false positive, true negative and false negative decisions, respectively, and *N* is the sample size [24].

The net benefit is defined as the benefit divided by the profit:

4 $$ NB=\frac{B}{p}=\frac{TP}{N}-\frac{l}{p}\frac{FP}{N} $$

In other words, the net benefit is the benefit that results from the normalization of the profit. In this aspect, the ‘loss-to-profit ratio’ is a weighting factor to give weight to the false positive decision compared to one unit of benefit of the true positive decision. It is important to note that profit and loss are unknown in most applications and it is impossible to measure them. This is common in medical decision making. One simply cannot measure or numerically anticipate the consequences of the true positive decision (the so-called profit of the operation, for instance) or the consequences of the false positive decision (for example, the loss of an unnecessary operation). That is why Vickers and Elkin suggested calculating this weighting factor (loss-to-profit ratio) as the odds of the threshold probability. The weights of the four possible outcomes (TP, FP, FN and TN) are not known; still, it is possible to make an acceptable assumption of the ‘loss’-to-‘profit’ ratio.

There is an important assumption by Vickers:

5 $$ \frac{l}{p}:= \frac{p_t}{1-{p}_t} $$

where *p*_*t*_ is the threshold probability (or cut-point), above which the outcome of a probability prediction model is labelled ‘positive’ and below which it is labelled ‘negative’ [22].

If we accept this assumption, net benefit simplifies to:

6 $$ NB=\frac{B}{p}=\frac{TP}{N}-\frac{p_t}{1-{p}_t}\frac{FP}{N} $$

The decision curve is a curve that illustrates the net benefit in function of the threshold level ***p***_***t***_. Using this method, we can compare the performance of different predictive models to show which model is more beneficial in function of the threshold probability. This method also shows the range of threshold levels where the decision is beneficial.

## Results

### Primary results for the predictors

Our investigations revealed that none of the predictors alone was sufficient for prediction. Candidate predictor PTV had AUC-ROC of 0.722 while AUC-ROC for BMI was 0.740, which is fair, but not sufficient for our purposes.

The best predictor was A_heart_, with AUC-ROC of 0.868. Table 1 shows the classification results of the most important candidate predictors and the primary results of the multivariate prediction models. These values in Table 1 are not cross-validated and based on data from 83 patients. Table 1 presents the model selection and the performance of the predictors alone.

**Table 1:** Classification results for the predictors and for the multivariate prediction models based on data from *n* = 83

Model	ROC-AUC	95% Confidence interval for ROC-AUC	Brier score
PTV (PTV candidate predictor only)	0.722	0.608, 0.836	0.213
BMI (BMI predictor only)	0.740	0.630, 0.850	0.201
Median distance (d_median_ predictor only)	0.787	0.690, 0.884	0.189
Area (A_heart_ predictor only)	0.868	0.791, 0.944	0.151
Logistic regression (main effect model, Model1)	0.906	0.854, 0.959	0.124
Logistic regression (forward LR selection model, Model2)	0.900	0.848, 0.953	0.132
Linear regression (Model3)	0.903	0.850, 0.957	0.139

Although it is possible to base recommendations on a single predictor value, our experience suggests that the predictive power of this simple approach is not satisfactory. This is why we constructed a multivariate prediction model.

### Multivariate models

There are several complex predictive methods in statistics, but our goal was to use a simple model with acceptable predictive performance.

Among the multivariate models, the backward likelihood ratio selection model was the ‘main effect model’: *area + BMI + median distance*. The forward likelihood ratio selection model was not a hierarchical model, as it contained two interaction terms: area*BMI + area*median distance without main effects.

Multiple linear regression seemed to be the most useful model with respect to the promising high AUC-ROC of 0.903 (results in Table 1) and the advantage noted above that the expected value of the LAD mean dose difference can also be calculated.

The results of the random cross-validation for the multiple regression model have a sensitivity of 80.7% and specificity of 87.5% [6].

### Results of comparing the models using decision curves

**Fig. 1:**
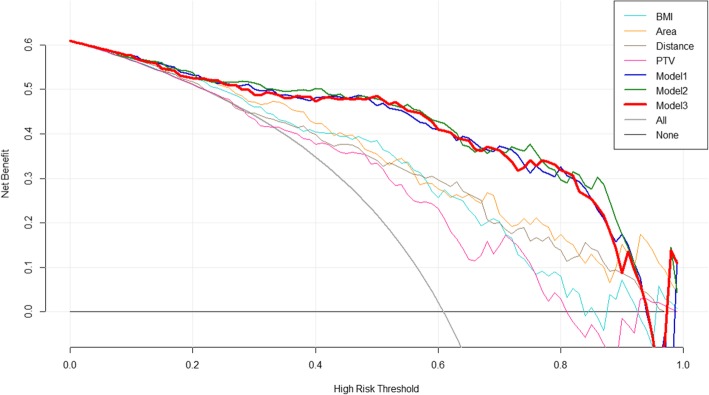
Decision curves for the four best predictors and three multivariate models. The vertical axis represents the value of net benefit, and the horizontal axis represents the threshold level (possible probability cut-points). Plotting net benefit in function of threshold level yields the decision curve. In the legend, PTV, Area, Distance and BMI refer to candidate predictor PTV, predictor A_heart_, predictor d_median_ and predictor BMI alone, respectively. Model1 to Model3 refer to the performance of the multivariate prediction models. Model2 is the main effect model (*area + BMI + median distance*), Model2 is the forward likelihood ratio selection model with two interaction terms (area*BMI + area*median distance), and Model3 is the linear regression-based model. This figure shows that the logistic regression models and the linear regression model lead to very similar high values of net benefit in a wide range of threshold levels and that none of the predictors alone can lead to similarly high values of net benefit

We examined the performance of the three best predictors alone with decision curves, and we compared them to the models described above. None of the best three predictors alone was comparable to the prediction models (Fig. 1).

The decision curves for the logistic regression model and the linear regression-based model were quite similar to each other. We can conclude that both models lead to high values of net benefit for a wide range of threshold probabilities. In other words, it is beneficial to use these models in respect of net benefit regardless of the current threshold probability. These results showed that these models can be used in clinical practice.

A bootstrap method was applied to construct 95% confidence intervals for the net benefit at various threshold levels for the main effect model [7]. 1000 bootstrap samples were generated by Microsoft Excel with a sample size of 138 each. Net benefit values were calculated by definition. The 2.5 and 97.5 percentiles of the net benefit values were calculated at each threshold level. The results are shown in Table 2.

**Table 2:** 95% confidence intervals for net benefit (logistic regression, main effect model) at different threshold levels. Confidence bounds are based on 1000 bootstrap samples

Threshold probability	Lower bound for net benefit	Upper bound for net benefit
0.1	0.564	0.573
0.2	0.496	0.547
0.3	0.450	0.527
0.4	0.423	0.517
0.5	0.391	0.507
0.6	0.319	0.467
0.7	0.256	0.435
0.8	0.210	0.435
0.9	0.000	0.333

The linear regression model is mathematically simple and can be very easily implemented (for instance, in Microsoft Excel). To the best of our knowledge, no similar composite models have been used to select treatment position in radiotherapy.

## Discussion

We presented different models based on patient-related parameters to predict the preferable treatment position in left-sided breast cancer radiotherapy and the mathematical aspects of the evaluation of the predictive power of these models. All the predictive models performed better than single predictors did, but the linear regression model was considered the most clinically relevant for quantitative estimation.

One merit of our study is its relatively large sample size and multivariate aspect. Decision curves and the AUC for the ROC results were found to be similar for the linear regression model and the two logistic regression models. Nevertheless, while the logistic regression models weight the outcomes on a binary scale, the linear regression model weights them in keeping with the magnitude of the difference. Since the linear regression model provides additional information, i.e. estimation of the dose difference, we decided to use the linear regression model. Knowledge of the estimated quantitative benefit of one or the other treatment position during radiotherapy may provide better guidance for the physician when considering various aspects, such as repositioning accuracy, patient comfort etc.

The application of AUC-ROC and measures like sensitivity and specificity is very common in radiotherapy planning, but we have not seen the approach of using decision curves in this field. Our investigations point to the clinical utility of predictive models.

There are certain limitations of the linear regression model we presented. The performance of the model is fair, but limited to a sensitivity of 80.7% and a specificity of 87.5%. These values seemed very stable throughout the different steps of the evaluation. Furthermore, in the next phase of development, very similar results were also found. In brief, a simple clinical tool which used the model was created and tested for clinical practice [7]. This tool estimates the difference of the expected dose values based on the BMI and the d_median_ and A_heart_ measured on a CT slice at the middle of the heart. The result was compared to that of the full CT series in both positions and the dosimetric data. The comparison revealed very consistent results from the simple tool and the original method (very similar sensitivity and specificity values) [7].

One limitation might be that we assumed a linear relationship between the predictors and the dependent variable. Our investigation revealed that none of the higher-order terms (squares or cubes of the predictors) improved the model at all. In other words, the linear relationship may be a target of criticism, but we found no other simple relationship more suitable for model building.

Zhao at el. built an SVM (support vector machine)-based two-step decision algorithm in a sample of 198 patients [11]. Their method is based on anatomical characteristics measured on a prone CT series. This classified patients into prone position radiotherapy or into another CT series in supine position for comparison. Although the numerical measures of the goodness of classification were impressive, that tool provided no numerical estimation of the advantage of one treatment position over the other; hence, no optimization could be practised. With their method, one only manage to filter out cases with an in-field heart volume over the acceptable threshold in the prone position and with the necessity of a second CT series in the supine position [11].

As noted earlier, although this report only discussed the use of the LAD mean dose difference as a primary outcome, there are other possible dependent variables, such as the heart dose. In our clinical practice, algorithm of the dose to the heart is also considered in a complex decision, but only as a secondary outcome measure [7]. In most centres, the most widely applied outcome measure is the mean heart dose [8, 25, 26]. Since a linear, non-threshold association exists between the mean heart dose and coronary events, mean heart dose may be regarded as an approximation of the doses to the LAD and other coronary arteries [25, 26]. Since there is a strong correlation between mean heart dose and LAD dose (R = 0.87 in both positions), we believe that the predictive model presented here could be adapted to local practice in any centre applying prone radiotherapy.

Moreover, the simple tool, which uses a single CT scan in the supine position, combined with the dose constraints described in everyday practice at the Department of Oncotherapy, University of Szeged, is satisfactory and of great utility. The linear regression-based model was also tested in a 28-case external dataset of left-side breast cancer patients from Liege and showed great consistency in our results noted above. Predicted treatment position was correct in 24 out of 28 (accuracy: 85.7%) cases [7].

## Conclusions

Our study has demonstrated that decision curves are useful in comparing our models. Any of the models could be implemented in clinical practice, but the linear regression model is the most useful and stable in facilitating the radiation treatment decision. In addition, this linear regression model is already implemented in everyday radiotherapy practice at the Department of Oncotherapy, University of Szeged, Szeged, Hungary.

## Data Availability

The datasets used and/or analysed during the current study are available from the corresponding author on reasonable request.

## Abbreviations

A_heart_: Area of the heart at the middle of the LAD
AUC: Area under curve
BMI: Body mass index
BS: Brier score
d_median_: Median distance between the LAD and the chest wall
FN: Number of false negative cases
FP: Number of false positive cases
LAD: Left anterior descending coronary artery
LR: Likelihood ratio
NB: Net benefit
OAR: Organ at risk
PTV: Planning target volume (operated breast with safety margin)
ROC: Receiver operating characteristic
TN: Number of true negative cases
TP: Number of true positive cases
T_p_: Threshold probability
wBS: Weighted Brier score
